# Spectrum of *PAH* gene variants among a population of Han Chinese patients with phenylketonuria from northern China

**DOI:** 10.1186/s12881-017-0467-7

**Published:** 2017-10-05

**Authors:** Ning Liu, Qiuying Huang, Qingge Li, Dehua Zhao, Xiaole Li, Lixia Cui, Ying Bai, Yin Feng, Xiangdong Kong

**Affiliations:** 1 Genetics and Prenatal Diagnosis Center, The First Affiliated Hospital of Zhengzhou University, Henan Engineering Research Center for Gene Editing of Human Genetic Disease, Jianshe Rd, Erqi District, Zhengzhou, Henan 450052 People’s Republic of China; 2 State Key Laboratory of Cellular Stress Biology, State Key Laboratory of Molecular Vaccinology and Molecular Diagnostics, Engineering Research Centre of Molecular Diagnostics, Ministry of Education, School of Life Sciences, Xiamen University, Xiangan Rd, Xiangan District, Xiamen, Fujian 361102 People’s Republic of China; 3 Neonatal Screening Center, The Third Affiliated Hospital of Zhengzhou University, Kangfu Rd, Erqi District, Zhengzhou, Henan 450052 People’s Republic of China; 4 Neonatal Screening Center, Zhengzhou Maternity and Child Care Hospital, Jinshui Rd, Jinshui District, Zhengzhou, Henan 450012 People’s Republic of China

**Keywords:** Phenylketonuria, Phenylalanine hydroxylase gene, *PAH* gene variant, MLPA

## Abstract

**Background:**

Phenylketonuria (PKU), which primarily results from a deficiency of phenylalanine hydroxylase (PAH), is one of the most common inherited inborn errors of metabolism that impairs postnatal cognitive development. The incidence of various PAH variations differs by race and ethnicity. The aim of the present study was to characterize the *PAH* gene variants of a Han population from Northern China.

**Methods:**

In total, 655 PKU patients and their families were recruited for this study; each proband was diagnosed both clinically and biochemically with phenylketonuria. Subjects were sequentially screened for single-base variants and exon deletions or duplications within *PAH* via direct Sanger sequencing and multiplex ligation-dependent probe amplification (MLPA).

**Results:**

A spectrum of 174 distinct *PAH* variants was identified: 152 previously documented variants and 22 novel variants. While single-base variants were distributed throughout the 13 exons, they were particularly concentrated in exons 7 (33.3%), 11 (14.2%), 6 (13.2%), 12 (11.0%), 3 (10.4%), and 5 (4.4%). The predominant variant was p.Arg243Gln (17.7%), followed by Ex6-96A > G (8.3%), p.Val399 = (6.4%), p.Arg53His (4.7%), p.Tyr356* (4.7%), p.Arg241Cys (4.6%), p.Arg413Pro (4.6%), p.Arg111* (4.4%), and c.442-1G > A (3.4%). Notably, two patients were also identified as carrying de novo variants.

**Conclusion:**

The composition of *PAH* gene variants in this Han population from Northern China was distinct from those of other ethnic groups. As such, the construction of a *PAH* gene variant database for Northern China is necessary to lay a foundation for genetic-based diagnoses, prenatal diagnoses, and population screening.

**Electronic supplementary material:**

The online version of this article (10.1186/s12881-017-0467-7) contains supplementary material, which is available to authorized users.

## Background

Phenylketonuria (PKU, #OMIM 261600), which exhibits autosomal recessive inheritance, is the most common inborn error of amino acid metabolism. PKU is caused by variations within the gene encoding phenylalanine hydroxylase (PAH), an enzyme that converts phenylalanine to other compounds, that result in the accumulation of phenylalanine (Phe) to neurotoxic levels [1]. Untreated PKU is associated with progressive intellectual impairment, accompanied by a constellation of additional symptoms, including eczematous rash, autism, seizures, and motor deficits. Hyperphenylalaninemia (HPA) comprises a group of genetically heterogeneous disorders, including deficiencies in PAH, tetrahydrobiopterin (BH4), and DNAJC12 [2], which are associated with six genes (*PAH*, *PTS*, *GCH1*, *QDPR*, *PCBD1*, and *DNAJC12*). Deficiency of the *GFRP* was so far not reported, and it is unclear if it would present with HPA as well. Notably, while PKU always causes HPA, not all cases of HPA are due to PKU. As timely screening and accurate diagnosis of HPA are important for treatment, *PAH* gene analysis is critical for the diagnosis, differential diagnosis, and correct treatment of this disorder.

The human *PAH* gene, which is located on chromosome 12q23.2, consists of 13 exons spanning 90 kb that encode a monomer protein of 452 amino acids. According to the open-access locus-specific database (LSD) (http://www.biopku.org/home/pah.asp), there are more than 900 known variants of this gene. As such, LSD is an invaluable resource of information for more efficient classification and management of patients [3].

Here we performed a comprehensive analysis of *PAH* gene variants compiled from 655 PKU patients and their families from Northern China. The primary aim of this study was to provide a basis for rapid and efficient genetic-based diagnosis, genetic counseling for the families of patients, and prenatal diagnosis of PKU in northern China.

## Methods

### Participants

A total of 655 unrelated Han families, in which at least one member was diagnosed with PKU, were recruited at the genetic counseling clinic of the First Affiliated Hospital of Zhengzhou University, the neonatal screening center of the Third Affiliated Hospital of Zhengzhou University, or Zhengzhou Maternity and Child Care Hospital between January 2008 and January 2016. The ancestral home of all subjects was in the Northern provinces of China. Patients with BH4 cofactor deficiency were excluded. Samples were collected via a newborn screening program. Each of the patients studied was diagnosed with HPA. Twelve patients were deceased when the parents came to the clinic, and the medical data for these individuals were integrated. The pretreatment plasma Phe level for each patient was >120 μmol/L (genotypes observed in patients with phenylketonuria and clinical phenotypes are provided in Additional file 1), as quantified in dried blood spots via the fluorescence test and tandem mass spectrometry. This study was approved by the Medical Ethics Committee of the First Affiliated Hospital of Zhengzhou University, and was performed according to the principles of the Declaration of Helsinki. All subjects or guardians provided signed informed consent.

### Genotype analysis

Peripheral blood samples were collected from the patients and parents in each of the 643 core families. For the 12 deceased-proband families, samples were collected only from the parents. Genomic DNA was extracted from each sample, and PCR primers were designed to amplify each of the 13 exons of the *PAH* gene, as well as their flanking introns, using previously published sequencing data [4]. PCR products were sequenced bi-directionally using an ABI 3130-xl gene analyzer (Life Technologies, Carlsbad, CA, USA). To identify nucleotide variations, sequences were aligned and inspected using a reference sequence from Ensembl (NM_000277). To determine sequence variability, variable sites in patient genes were aligned with the corresponding sites from the respective parents. We excluded non-biological relationships in de novo-variant pedigrees using the Promega PowerPlex 21 HS system (Promega Corporation, Madison, WI, USA).

### Multiplex ligation-dependent probe amplification (MLPA) analysis

DNA samples harboring one or no variant(s), as determined by sequencing, were confirmed and assayed separately for *PAH* copy-number variants by MLPA analysis using a PAH MLPA kit (SALSA P055; MRC-Holland, Amsterdam, The Netherlands), according to the manufacturer’s protocol. Amplification products were separated using an ABI 3130-xl Genetic Analyzer. Raw data were analyzed using GeneMapper® Software Version 4.2 to estimate the size of the PCR products and to obtain peak areas. Height ratios of fluorescent peaks that were lower than the normal height ratio (0.7) were indicative of the presence of exon deletions.

### Nomenclature and variation validation

Previously characterized pathogenic variants were identified by comparison with those deposited in disease databases, including the Human Gene Mutation Database (HGMD) and BIOPKUdb. Single nucleotide polymorphisms (SNPs) were further excluded by querying the 1000 Genomes Data (http://www.1000genomes.org/), dbSNP, and HapMap databases. Novel variants were named according to the international gene mutation nomenclature system (http://www.HGVS.org/varnomen). The pathogenic effects associated with variations of interest were calculated using prediction tool algorithms (e.g., PROVEAN, PolyPhen-2, and MutationTaster).

## Results

### *PAH* gene variant spectrum

In this study, potential disease-causing mutations were identified in 1266 of the 1310 independent alleles tested (detection rate = 96.6%). A total of 613 (93.6%) patients were completely genotyped. Among the fully genotyped patients, 534 (81.5%) carried compound heterozygous variants, 63 (9.6%) carried homozygous variants, 40 (6.1%) harbored a single heterozygous variant, and 16 (2.4%) harbored three separate variants. Notably, however, we failed to detect variant alleles in two (0.3%) patients.

A spectrum of 174 distinct *PAH* gene variants was detected in the 655 PKU families recruited in this study (high frequency and novel variants are summarized in Table 1, while the complete list of variants is provided in Additional file 2). These variants fell into seven categories: missense variants (107, 61.4%), splicing variants (34, 19.4%), nonsense variants (16, 9.2%), small deletions (10, 5.7%), large deletions (6, 3.4%), insertion variants (1, 0.6%), and indel variants (1, 0.6%).

**Table 1:** High frequency variants of the *PAH* gene

Number	Trivial name (Protein effect)	Systematic name (DNA level)	Location	Variant type	Allele frequency (%)
1	p.Arg53His	c.158G > A	Exon 2	Missense	4.7
2	p.Arg111*	c.331C > T	Exon 3	Nonsense	4.4
3	—	c.442-1G > A	Intron 4	Splicing	3.4
4	p.Ex6-96A > G	c.611A > G	Exon 6	Splicing	8.3
5	p.Arg241Cys	c.721C > T	Exon 7	Missense	4.6
6	p.Arg243Gln	c.728G > A	Exon 7	Missense	17.7
7	p.Tyr356*	c.1068C > A	Exon 11	Nonsense	4.7
8	p.Val399=	c.1197A > T	Exon 11	Splicing	6.4
9	p.Arg413Pro	c.1238G > C	Exon 12	Missense	4.6

p.Arg243Gln (17.7%) was the most prevalent variant, followed by Ex6­96A > G (8.3%), p.Val399 = (6.4%), p.Arg53His (4.7%), p.Tyr356* (4.7%), p.Arg241Cys (4.6%), p.Arg413Pro (4.6%), p.Arg111* (4.4%), and c.442-1G > A (3.4%). These nine variants accounted for 58.7% of all variant alleles detected.

The 174 distinct variants were distributed throughout the 13 exons and flanking intron regions of the *PAH* gene; however, the largest number of variants was observed in exon 7 and its flanking intron regions (33.3%, 423/1269), followed by exon 11 (14.4%, 183/1269), exon 6 (13.1%, 166/1269), exon 12 (10.9%, 138/1269), exon 3 (10.4%, 132/1269), and exon 5 (4.4%, 56/1269) (Table 2).

**Table 2:** Exon Distribution and allelic frequencies of *PAH* gene

Location (exon + intron)	No. of mutation kinds	No. of mutation allele	Frequency of mutation allele(%)
1	2	3	0.24
2	6	66	5.2
3	13	134	10.48
4	7	54	4.33
5	18	56	4.41
6	24	166	13.24
7	34	423	33.25
8	6	8	0.63
9	5	10	0.79
10	16	26	2.05
11	21	183	14.18
12	17	138	11.03
13	2	2	0.16

### MLPA analysis

Of the 53 PKU patients subjected to MLPA genotyping analysis, 13 contained exon deletions. Specifically, we detected a deletion spanning the 5′-UTR and exon 1 in eight patients, a deletion of exon 3 in one patient, a deletion of exon 5 in one patient, a deletion of exons 4 and 5 in two patients, and a deletion of exons 4–7 in one patient.

### De novo variants pedigrees

By screening the corresponding variant gene sites of the parents, we found that two patients carried de novo variants. Paternity testing was subsequently performed to confirm the biological nature of the relationship between the patient and parent. Meanwhile, the identification of three distinct variants in 16 families confirmed that the variants had originated from the parents, with one of the parents carrying two variants on the same allele.

### Novel sequence variants

Twenty-two novel variants that have not been registered in the BIOPKU database were identified in this research: IVS4-14C > T, IVS8 + 16 T > A, IVS10-13delT, IVS11-3 T > G, p.Tyr154*, p.Tyr268*, p.Arg155Valfs*40, p.Ser231Valfs*52, p.Leu194Glufs*6, p.Asp75His, p.Ile94Val, p.Gly188Val, p.Cys203Ser, p.Leu227Val, p.Glu228Asp, p.Ser250Phe, p.Ser310Cys, p.Ser339Phe, p.Lys341Asn, p.Pro362Ser, p.Pro366Ala, and p.Leu444Phe. The predicted biological effects of these novel variants are listed in Table 3: 19 of the variations detected, particularly the missense, nonsense, and frame-shift variants, had the potential to be damaging, deleterious, and disease causing. Conversely, the three splicing variants IVS4-14C > T, IVS8 + 16 T > A, and IVS10-13delT were found to comprise polymorphisms by MutationTaster, and could not be predicted using the other two tools.

**Table 3:** Pathological analysis of the 22 novel variants of the *PAH* gene detected in this study

NO.	Mutation type	Trivial name (Protein effect)	Systematic name (DNA level)	PolyPhen-2	PROVEAN	MutationTaster
1	Splice	IVS4-14C > T	c.442-14 C > T			Polymorphism
2		IVS8 + 16 T > A	c.912 + 16 T > A			Polymorphism
3		IVS10-13delT	c.1066-13delT			Polymorphism
4		IVS11-3 T > G	c.1200-3 T > G			Disease-causing
5		p.Tyr154*	c.462C > A			Disease-causing
6	Nonsense	p.Tyr268*	c.804C > A			Disease-causing
7		p.Arg155Valfs*40	c.463delC			Disease-causing
8	Frameshift	p.Ser231Valfs*52	c.690-691insG			Disease-causing
9		p.Leu194Glufs*6	c.580C > GA			Disease-causing
10		p.Asp75His	c.223 G > C	Damaging	Neutral	Disease-causing
11		p.Ile94Val	c.280 A > G	Benign	Neutral	Disease-causing
12		p.Gly188Val	c.563G > T	Damaging	Deleterious	Disease-causing
13		p.Cys203Ser	c.607 T > A	Damaging	Deleterious	Disease-causing
14		p.Leu227Val	c.679C > G	Damaging	Deleterious	Disease-causing
15	Missense	p.Glu228Asp	c.684A > C	Benign	Neutral	Disease-causing
16		p.Ser250Phe	c.749C > T	Damaging	Deleterious	Disease-causing
17		p.Ser310Cys	c.929C > G	Damaging	Deleterious	Disease-causing
18		p.Ser339Phe	c.992 T > C	Damaging	Deleterious	Disease-causing
19		p.Lys341Asn	c.1023G > C	Damaging	Deleterious	Disease-causing
20		p.Pro362Ser	c.1084 C > T	Damaging	Deleterious	Disease-causing
21		p.Pro366Ala	c.1096 C > G	Benign	Deleterious	Disease-causing
22		p.Leu444Phe	c.1330 C > T	Damaging	Deleterious	Disease-causing

## Discussion

The distribution of PKU among the Chinese population shows geographical and ethnic differences. While the overall incidence of PKU in China is 1/10,000–1/16,000, corresponding to a carrier frequency of approximately 1 in 50 [5], the prevalence varies considerably throughout the country, with much higher rates in Northern China (1/3425–1/7849) than in Southern China [6–9]. Investigation of the characteristics of *PAH* gene variants in different populations is very important for early, rapid, and accurate genetic-based diagnosis and subsequent patient treatment. Moreover, characterization of the distribution of *PAH* gene variants between populations provides important information regarding the ethnic migration and evolution of humans.

In our study, p.Arg243Gln (17.7%), followed by Ex6-96A > G, p.Val399=, p.Arg53His, p.Tyr356*, p.Arg241Cys, p.Arg413Pro, p.Arg111*, and c.442-1G > A, were the most prevalent variants, respectively. Indeed, these nine variants accounted for two-thirds of all those identified. These results were consistent with those of a previous study, although the rank order of these mutations was different [10–13]. Notably, previous molecular studies examining the spectra of variations in PKU patients in Asian populations indicated that, in general, mutations were not randomly distributed, and that certain variations show regional associations. For comparison, variants p.Arg413Pro, c.442-1G > A, p.Arg241Cys, p.Arg243Gln, p.Thr278Ile, Ex6-96A > G, p.Tyr356*, and p.Arg111* accounted for approximately 74.4% of the PKU in a population of Japanese patients [14], while p.Arg243Gln, c.442-1G > A, and Ex6-96A > G were the most common variants in Korean PKU patients [15]. In this study, variants were distributed throughout the entire *PAH* gene; however, the most commonly affected regions were exons 6, 7, and 11. Indeed, *PAH* gene variants appear to be concentrated in exons 7, 6, 11, 5, 12, 10, and 3, respectively, among Asian populations [11–15]. The similarities in the variant spectra of Chinese, Korean, and Japanese populations suggest that human migration, fusion, and evolution in these three countries were similar. In contrast, significant differences are found with regard to gene variants between Western and Eastern countries [16]. Furthermore, consistent with trends observed in Asian countries, *PAH* gene variants among European countries were found to exhibit regionality.

*PAH* gene variants in the Chinese population were predominant in specific exons, and hot spot variants were observed. Based on genetic research of Chinese people [10–13, 17–19], we propose a strategy for *PAH* gene screening in Northern Chinese populations. First, exons 3, 5, 6, 7, 11, and 12 should be preferentially Sanger sequenced, followed by sequencing of the other exons. After Sanger sequencing, patients lacking at least one identified variant should be examined via MLPA to screen for exon deletions/duplications within *PAH*. Lastly, if patients presenting with high phenylalanine levels cannot be diagnosed by *PAH* gene analysis, next-generation sequencing of genes related to BH4 deficiency, including *PTS, GCH1, PCBD1*, *QDPR*, and *GFRP*, should be applied. High-throughput automated sequencing techniques offer promise for revolutionizing the molecular diagnosis of PKU and BH4 disorders [20, 21]. Our understanding of the molecular basis of PKU has increased dramatically in recent years, driven largely by the availability of ever-more powerful techniques for analyzing and visualizing the effects of mutations on proteins [16].

A notable finding of this study was that 16 patients harbored three *PAH* gene variants, each of which were previously identified as pathogenic. Further analysis revealed that two variants were derived from one of the parents. However, no relevant PKU symptoms were observed in the parents carrying the two variants, suggesting that these two variants are located on the same allele. In Turkey, Dobrowolski et al. reported 588 cases of PKU or high levels of phenylalanine in nine patients carrying three or more pathogenic variants [22]. In addition, Okano identified nine individuals harboring the p.Arg53His variant among 203 Japanese PKU patients [23]. In our study, the p.Arg53His variant was identified as acting in cis with c.842 + 2 T > A in nine patients; however, because of the small number of cases, unbalanced variants between these patients cannot be confirmed. The results of in vitro expression experiments demonstrated that a p.Arg53His-type PAH enzyme retains approximately 79% of wild-type activity [24], with a smaller effect on activity being observed when co-existing with other variants. In a previous study, Gu and Wang suggested that p.Arg53His is associated with a milder form of HPA; however, it cannot be discounted that this variant comprises a SNP among healthy populations [25]. Therefore, in future work, uncovering the effects of *PAH* gene variants on PAH protein function is essential for clarifying the nature of this variant.

Interestingly, there were two instances in which PKU pedigrees identified potential de novo *PAH* gene variants (i.e., patient exhibited compound heterozygous variants despite one of the parents not carrying one of the variations). After excluding a non-biological relationship between parent/child, we considered these to be true de novo variants. As the sequence of *PAH* is relatively conserved in the human genome, the incidence rate of de novo variants is very low. Indeed, such de novo variants have yet to be reported. Thus, the identification of de novo variants may permit accurate and rational genetic counseling for these families.

## Conclusions

**Fig. 1:**
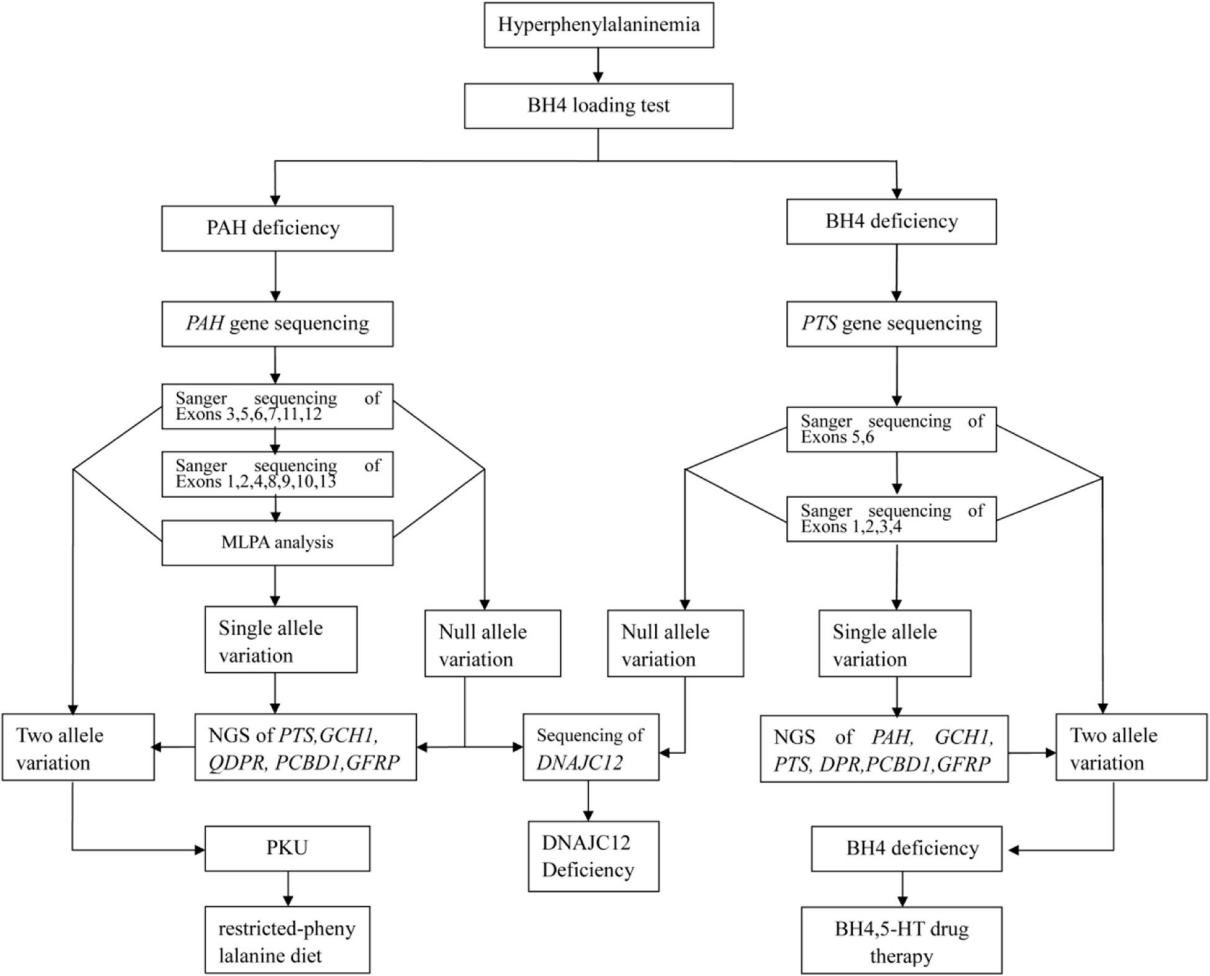
Flow chart depicting the strategy used for gene diagnosis in hyperphenylalaninemia patients

We presented a comprehensive and systematic analysis of *PAH* variants in 655 Chinese patients with PKU. We obtained a *PAH* gene variant spectrum for the Northern Chinese population and devised a strategy for gene diagnosis using PKU pedigrees (Fig. 1). Our findings will provide for rapid and efficient genetic-based diagnosis, genetic counseling, and prenatal diagnosis of PKU in China, particularly in the northern regions. Further explorations of the relationship between genotype and phenotype, as well as the consequences of gene variants are crucial. Locus-specific and genotype databases are today an invaluable resource of information for more efficient classification and management of patients.

## Additional files

Additional file 1: Genotype-phenotype correlations in patients with phenylketonuria. This file contains a description of the basic information, pretreatment plasma Phe level, clinical phenotype, and the corresponding genotypes for each patient. (XLS 106 kb)

Additional file 2: Spectrum of *PAH* gene variants in a Chinese Han population. This file contains the variation spectrum in the Han Chinese population, allele frequencies, and variation characteristics. (DOCX 31 kb)

## Abbreviations

BH4: Tetrahydrobiopterin
HPA: Hyperphenylalaninemia
MLPA: Multiplex ligation-dependent probe amplification
PAH: Phenylalanine hydroxylase
PKU: Phenylketonuria
