# Correction to: Spectrum of *PAH* gene variants among a population of Han Chinese patients with phenylketonuria from northern China

**DOI:** 10.1186/s12881-017-0516-2

**Published:** 2018-01-09

**Authors:** Ning Liu, Qiuying Huang, Qingge Li, Dehua Zhao, Xiaole Li, Lixia Cui, Ying Bai, Yin Feng, Xiangdong Kong

**Affiliations:** 1 Genetics and Prenatal Diagnosis Center, The First Affiliated Hospital of Zhengzhou University, Henan Engineering Research Center for Gene Editing of Human Genetic Disease, Jianshe Rd, Erqi District, Zhengzhou, Henan 450052 People’s Republic of China; 2 State Key Laboratory of Cellular Stress Biology, State Key Laboratory of Molecular Vaccinology and Molecular Diagnostics, Engineering Research Centre of Molecular Diagnostics, Ministry of Education, School of Life Sciences, Xiamen University, Xiangan Rd, Xiangan District, Xiamen, Fujian 361102 People’s Republic of China; 3 Neonatal Screening Center, The Third Affiliated Hospital of Zhengzhou University, Kangfu Rd, Erqi District, Zhengzhou, Henan 450052 People’s Republic of China; 4 Neonatal Screening Center, Zhengzhou Maternity and Child Care Hospital, Jinshui Rd, Jinshui District, Zhengzhou, Henan 450012 People’s Republic of China

## Correction

Following publication of the original article [1], the authors reported an error in Table 3 on page 4. Variant No. 18 should be “p.Ser339Phe c.1016C>T” (as given in Number 117 of Additional file 2). Other errors were found in the original Additional file 2, which has been replaced with an updated version (see below).

Additional file 2. Spectrum of *PAH* gene variants in a Chinese Han population

**Table:** 

Number	Trivial name (Protein effect)	Systematic name (DNA level)	Location	Variant type	Allele frequency (%)
1	p.S16*	c.47-48delCT	Exon 1	Deletion	0.2
2	—	c.61-3T>C	Intron 1	Splicing	0.1
3	p.Arg53His	c.158G>A	Exon 2	Missense	4.7
4	p.Arg53Cys	c.157C>T	Exon 2	Missense	0.1
5	p.Glu44del	c.131-133delAAG	Exon 2	Deletion	0.2
6	p.Glu56Asp	c.168G>T	Exon 2	Missense	0.1
7	—	c.168+2T>C	Intron 2	Splicing	0.1
8	—	c.168+5G>C	Intron 2	Splicing	0.1
9	p.Ile65Thr	c.194T>C	Exon 3	Missense	0.5
10	p.Ile65Ser	c.194T>G	Exon 3	Missense	0.2
11	p.Ser70del	c.208-210delTCT	Exon 3	Deletion	2.5
12	**p.Asp75His** ^△^	c.223G>C	Exon 3	Missense	0.1
13	p.Tyr77*	c.231T>G	Exon 3	Nonsense	0.1
14	**p.Ile94Val** ^△^	c.280A>G	Exon 3	Missense	0.1
15	p.Asp101Asn	c.301G>A	Exon 3	Missense	0.3
16	p.Gly103Asp	c.308G>A	Exon 3	Missense	0.2
17	p.His107Arg	c.320A>G	Exon 3	Missense	1.8
18	p.Arg111*	c.331C>T	Exon 3	Nonsense	4.4
19	p.Glu78Phefs*13	c.232-235delGAAT	Exon 3	Deletion	0.1
20	p.Ile95del	c.284-286deTCA	Exon 3	Deletion	0.1
21	IVS3-2A>G	c.353-2A>G	Intron3	Splicing	0.1
22	p.Pro147Leu	c.440C>T	Exon 4	Missense	0.2
23	—	c.441+1G>A	Intron 4	Splicing	0.1
24	—	c.441+3G>C	Intron 4	Splicing	0.4
25	—	c.442-1G>A	Intron 4	Splicing	3.4
26	—	c.442-1G>C	Intron 4	Splicing	0.1
27	—	**c.442-14C>T** ^△^	Intron 4	Splicing	0.1
28	p.Tyr154*^△^	c.462C>A	Exon 5	Nonsense	0.1
29	p.Arg155His	c.464G>A	Exon 5	Missense	0.2
30	p.Arg156Pro	c.466G>C	Exon 5	Missense	0.2
31	p.Arg158Trp	c.472C>T	Exon 5	Missense	0.5
32	p.Arg158Gln	c.473G>A	Exon 5	Missense	0.5
33	p.Gln160*	c.478 C>T	Exon 5	Nonsense	0.2
34	p.Phe161Ser	c.482T>C	Exon 5	Missense	1.0
35	p.Ala165Asp	c.494C>A	Exon 5	Missense	0.1
36	p.Tyr166*	c.498C>G	Exon 5	Nonsense	0.6
37	p.Arg169Ser	c.505C>A	Exon 5	Missense	0.1
38	p.Arg169Cys	c.505C>T	Exon 5	Missense	0.2
39	p.Arg169His	c.506G>A	Exon 5	Missense	0.1
40	p.His170Arg	c.509A>G	Exon 5	Missense	0.1
41	p.His170Gln	c. 510T>A	Exon 5	Missense	0.3
42	p.Arg155Valfs*40 ^△^	c.463delC	Exon 5	Deletion	0.1
43	—	c.509+1G>A	Intron 5	Splicing	0.1
44	—	c.510-1G>A	Intron 5	Splicing	0.1
45	—	c.510-1G>C	Intron 5	Splicing	0.1
46	p.Gly171Arg	c.511G>A	Exon 6	Missense	0.1
47	p.Gln172His	c.516G>T	Exon 6	Missense	0.1
48	p.Arg176*	c.526C>T	Exon 6	Nonsense	2.0
49	p.Glu178Lys	c.532 G>A	Exon 6	Missense	0.1
50	p.Glu183Gly	c.548A>G	Exon 6	Missense	0.1
51	p.Trp187*	c.561G>A	Exon 6	Nonsense	0.1
52	p.Trp187Arg	c.559T>C	Exon 6	Missense	0.1
53	**p.Gly188Val** ^△^	c.563G>T	Exon 6	Missense	0.1
54	p.His201Arg	c.602A>G	Exon 6	Missense	0.1
55	**p.Cys203Ser** ^△^	c.607T>A	Exon 6	Missense	0.1
56	p. Ex6-96A>G	c.611A>G	Exon 6	Splicing	0.1
57	p.Cys217Tyr	c.650G>A	Exon 6	Missense	0.1
58	p.Ile224Thr	c.671T>C	Exon 6	Missense	0.4
59	**p.Leu227Val** ^△^	c.679C>G	Exon 6	Missense	0.1
60	**p.Glu228Asp** ^△^	c.684A>C	Exon 6	Missense	0.1
61	p.Val230Ile	c.688G>A	Exon 6	Missense	0.5
62	p.Val230A	c.689T>C	Exon 6	Missense	0.1
63	p.Gln232*	c.694C>T	Exon 6	Nonsense	0.2
64	p.Phe233Leu	c.699C>A	Exon 6	Missense	0.2
65	p.Leu194Glufs*6 ^△^	c.580C>GA	Exon 6	Indel	0.1
66	p.Ser231Valfs*52 ^△^	c.690-691insG	Exon 6	Insertion	0.1
67	—	c.707-1G>A	Intron 6	Splicing	0.2
68	p.Arg241Cys	c.721C>T	Exon 7	Missense	4.6
69	p.Arg241His	c.722G>A	Exon 7	Missense	0.4
70	p.Arg241Leu	c.722G>T	Exon 7	Missense	0.1
71	p.Leu242Phe	c.724C>T	Exon 7	Missense	0.2
72	p.Arg243Gln	c.728G>A	Exon 7	Missense	17.7
73	p.Arg243*	c.727C>T	Exon 7	Nonsense	0.1
74	p.Val245Met	c.733G>A	Exon 7	Missense	0.1
75	p.Gly247Val	c.740G>T	Exon 7	Missense	1.5
76	p.Gly247Arg	c.739G>C	Exon 7	Missense	0.7
77	**p.Ser250Phe** ^△^	c.749C>T	Exon 7	Missense	0.1
78	p.Arg252Gln	c.755G>A	Exon 7	Missense	0.6
79	p.Arg252Trp	c.754C>T	Exon 7	Missense	0.3
80	p.Arg252Gly	c. 754C>G	Exon 7	Missense	0.1
81	p.Arg252Pro	c.755G>C	Exon 7	Missense	0.1
82	p.Leu255Ser	c.764T>C	Exon 7	Missense	0.4
83	p.Gly257Val	c.770G>T	Exon 7	Missense	0.6
84	p.Arg261Gln	c.782G>A	Exon 7	Missense	1.7
85	p.Arg261*	c.781C>T	Exon 7	Nonsense	0.1
86	p.Phe263Leu	c.787T>C	Exon 7	Missense	0.1
87	p.Gln267Glu	c.799C>G	Exon 7	Missense	0.2
88	p.Tyr268* ^△^	c.804C>A	Exon 7	Nonsense	0.1
89	p.Arg270Lys	c.809G>A	Exon 7	Missense	0.2
90	p.Arg270Ile	c. 809G>T	Exon 7	Missense	0.1
91	p.His271Arg	c.812A>G	Exon 7	Missense	0.1
92	p.Pro275Leu	c.824C>T	Exon 7	Missense	0.2
93	p.Met276Lys	c.827T>A	Exon 7	Missense	0.2
94	p.Met276Arg	c.827T>G	Exon 7	Missense	0.1
95	p.Thr278Ile	c.833C>T	Exon 7	Missense	0.1
96	p.Glu280Lys	c.838G>A	Exon 7	Missense	0.4
97	p.Pro281Arg	c.842C>G	Exon 7	Missense	0.1
98	p.Arg241Profs*100	c.722delG	Exon 7	Deletion	0.5
99	—	c.842+1G>A	Intron 7	Splicing	0.2
100	—	c.843-1G>A	Intron 7	Splicing	0.2
101	—	c.842+2T>A	Intron 7	Splicing	1.5
102	p.Val291Met	c.871G>A	Exon 8	Missense	0.1
103	p.Ala300Ser	c.898G>T	Exon 8	Missense	0.1
104	p.Ser303Pro	c.907T>C	Exon 8	Missense	0.1
105	p.Ser303Profs*38	c.907delT	Exon 8	Deletion	0.1
106	—	c.912+1G>A	Intron 8	Splicing	0.1
107	—	**c.912+16T>A** ^△^	Intron 8	Splicing	0.1
108	—	c.913-7A>G	Intron 8	Splicing	0.2
109	p.Ser310Phe	c.929C>T	Exon 9	Missense	0.1
110	**p.Ser310Cys** ^△^	c.929C>G	Exon 9	Missense	0.1
111	p.Gly312Val	c.935G>T	Exon 9	Missense	0.1
112	p.Pro314Thr	c.940C>A	Exon 9	Missense	0.4
113	p.Ala322Thr	c.964G>A	Exon 9	Missense	0.2
114	p.Ile324Asn	c.971T>A	Exon 10	Missense	0.4
115	p.Trp326*	c.977G>A	Exon 10	Nonsense	0.2
116	p.Phe331Ser	c.992T>C	Exon 10	Missense	0.1
117	**p.Ser339Phe** ^△^	c.1016C>T	Exon 10	Missense	0.1
118	**p.Lys341Asn** ^△^	c.1023G>C	Exon 10	Missense	0.1
119	p.Ala342Hisfs*58	c.1024delG	Exon 10	Deletion	0.1
120	p.Gly344Asp	c.1031G>A	Exon 10	Missense	0.1
121	p.Gly344Ser	c.1030G>A	Exon 10	Missense	0.1
122	p.Ala345Thr	c.1033G>A	Exon 10	Missense	0.1
123	p.Ser349Ala	c.1045T>G	Exon 10	Missense	0.4
124	p.Gly352Arg	c.1054G>C	Exon 10	Missense	0.1
125	p.Gln355*	c.1063C>T	Exon 10	Nonsense	0.1
126	—	c.1066-1G>T	Intron 10	Splicing	0.2
127	—	c.1066-11G>A	Intron 10	Splicing	0.1
128	—	**c.1066-13delT** ^△^	Intron 10	Splicing	0.1
129	—	c.1066-14C>G	Intron 10	Splicing	0.1
130	p.Tyr356*	c.1068C>A	Exon 11	Nonsense	4.7
131	p.Cys357*	c.1071C>A	Exon 11	Nonsense	0.1
132	**p.Pro362Ser** ^△^	c.1084C>T	Exon 11	Missense	0.1
133	p.Pro362Thr	c.1084C>A	Exon 11	Missense	0.1
134	p.Lys363Asn	c.1089G>T	Exon 11	Missense	0.2
135	**p.Pro366Ala** ^△^	c.1096C>G	Exon 11	Missense	0.1
136	p.Thr372Ser	c.1114A>T	Exon 11	Missense	0.1
137	p.Thr372Arg	c.1115 C>G	Exon 11	Missense	0.1
138	p.Ala373Thr	c.1117G>A	Exon 11	Missense	0.1
139	p.Gln375Glu	c.1123C>G	Exon 11	Missense	0.3
140	p.Thr380Met	c.1139C>T	Exon 11	Missense	0.2
141	p.Val388Met	c.1162G>A	Exon 11	Missense	0.1
142	p.Ser391Thr	c.1172G>C	Exon 11	Missense	0.1
143	p.Phe392Ile	c.1174T>A	Exon 11	Missense	0.4
144	p.Lys398=	c.1194A>G	Exon 11	Splicing	0.1
145	p.Val399=	c.1197A>T	Exon 11	Splicing	6.4
146	p.Arg400Thr	c.1199G>C	Exon 11	Missense	0.5
147	p.Arg400Lys	c.1199G>A	Exon 11	Missense	0.2
148	—	c.1199+1G>C	Intron11	Splicing	0.2
149	—	c.1199+2T>C	Intron 11	Splicing	0.2
150	—	c.1200-1G>C	Intron 11	Splicing	0.1
151	—	c.1200-1G>A	Intron 11	Splicing	0.2
152	—	**c.1200-3T>G** ^△^	Intron11	Splicing	0.2
153	p.Ala403Val	c.1208C>T	Exon 12	Missense	1.0
154	p.Arg408Trp	c.1222C>T	Exon 12	Missense	0.4
155	p.Arg408Gln	c.1223G>A	Exon 12	Missense	0.6
156	p.Arg413Pro	c.1238G>C	Exon 12	Missense	4.6
157	p.Tyr414*	c.1242C>A	Exon 12	Nonsense	0.1
158	p.Asp415Tyr	c.1243G>T	Exon 12	Missense	0.1
159	p.Thr418Pro	c.1252A>C	Exon 12	Missense	0.6
160	p.Gln419Arg	c.1256A>G	Exon 12	Missense	0.7
161	p.Ile421Thr	c.1262T>C	Exon 12	Missense	0.2
162	p.Gln429Lys	c.1285C>A	Exon 12	Missense	0.1
163	p.Leu430Pro	c.1289T>C	Exon 12	Missense	0.2
164	p.Ala434Asp	c.1301C>A	Exon 12	Missense	1.7
165	—	c.1315+4A>G	Intron 12	Splicing	0.1
166	—	c.1315+6T>A	Intron 12	Splicing	0.3
167	—	c.1316-2A>C	Intron 12	Splicing	0.2
168	**p.Leu444Phe** ^△^	c.1330C>T	Exon 13	Missense	0.1
169	p.*453Proext*33	c.1357delTAAAG	Exon 13	Deletion	0.1
170			5’ UTR ~E1	Deletion	0.6
171	c.(168+1_169-1)_c. (352+1_353-1)del		E3	Deletion	0.1
172	c. (441+1_442-1)_(842+1_843-1)del		E4~E7	Deletion	0.1
173	c. (441+1_442-1) _c. (509+1_510-1)del		E5	Deletion	0.1
174	c. (441+1_442-1)_(509+1_510-1)del		E4~E5	Deletion	0.2
Detected					96.6
Unknown					3.4
Total					100

^△^Novel alleles identified in the current study.
