# Supplementary material for: Spectrum of PAH gene variants among a population of Han Chinese patients with phenylketonuria from northern China
Source: BMC Med Genet. 2017 Oct 5;18:108. doi: 10.1186/s12881-017-0467-7 (PMC5629770; doi:10.1186/s12881-017-0467-7)
Supplement: Supplementary file 2 — Spectrum of PAH gene variants in a Chinese Han population. This file contains the variation spectrum in the Han Chinese population, allele frequencies, and variation characteristics. (DOCX 31 kb) [file 12881_2017_467_MOESM2_ESM.docx]

**Additional file 2.** **Spectrum of *PAH* gene variants in a Chinese Han population**

| Number | Trivial name  (Protein effect) | Systematic name  (DNA level) | Location | Variant type | Allele frequency  (%) |
| --- | --- | --- | --- | --- | --- |
|  | p.S16* | c.47-48delCT | Exon 1 | Deletion | 0.2 |
|  | — | c.61-3T>C | Intron 1 | Splicing | 0.1 |
|  | p.Arg53His | c.158G>A | Exon 2 | Missense | 4.7 |
|  | p.Arg53Cys | c.157C>T | Exon 2 | Missense | 0.1 |
|  | p.Glu44del | c.131-133delAAG | Exon 2 | Deletion | 0.2 |
|  | p.Glu56Asp | c.168G>T | Exon 2 | Missense | 0.1 |
|  | — | c.168+2T>C | Intron 2 | Splicing | 0.1 |
|  | — | c.168+5G>C | Intron 2 | Splicing | 0.1 |
|  | p.Ile65Thr | c.194T>C | Exon 3 | Missense | 0.5 |
|  | p.Ile65Ser | c.194T>G | Exon 3 | Missense | 0.2 |
|  | p.Ser70del | c.208-210delTCT | Exon 3 | Deletion | 2.5 |
|  | **p.Asp75His**^△^ | c.223G>C | Exon 3 | Missense | 0.1 |
|  | p.Tyr77* | c.231T>G | Exon 3 | Nonsense | 0.1 |
|  | **p.Ile94Val**^△^ | c.280A>G | Exon 3 | Missense | 0.1 |
|  | p.Asp101Asn | c.301G>A | Exon 3 | Missense | 0.3 |
|  | p.Gly103Asp | c.308G>A | Exon 3 | Missense | 0.2 |
|  | p.His107Arg | c.320A>G | Exon 3 | Missense | 1.8 |
|  | p.Arg111* | c.331C>T | Exon 3 | Nonsense | 4.4 |
|  | p.Glu78Phefs*13 | c.232-235delGAAT | Exon 3 | Deletion | 0.1 |
|  | p.Ile95del | c.284-286deTCA | Exon 3 | Deletion | 0.1 |
|  | IVS3-2A>G | c.353-2A>G | Intron3 | Splicing | 0.1 |
|  | p.Pro147Leu | c.440C>T | Exon 4 | Missense | 0.2 |
|  | — | c.441+1G>A | Intron 4 | Splicing | 0.1 |
|  | — | c.441+3G>C | Intron 4 | Splicing | 0.4 |
|  | — | c.442-1G>A | Intron 4 | Splicing | 3.4 |
|  | — | c.442-1G>C | Intron 4 | Splicing | 0.1 |
|  | — | **c.442-14C>T**^△^ | Intron 4 | Splicing | 0.1 |
|  | p.Pro147Leu^△^ | c.462C>A | Exon 5 | Nonsense | 0.1 |
|  | p.Arg155His | c.464G>A | Exon 5 | Missense | 0.2 |
|  | p.Arg156Pro | c.466G>C | Exon 5 | Missense | 0.2 |
|  | p.Arg158Trp | c.472C>T | Exon 5 | Missense | 0.5 |
|  | p.Arg158Gln | c.473G>A | Exon 5 | Missense | 0.5 |
|  | p.Gln160* | c.478 C>T | Exon 5 | Nonsense | 0.2 |
|  | p.Phe161Ser | c.482T>C | Exon 5 | Missense | 1.0 |
|  | p.Ala165Asp | c.494C>A | Exon 5 | Missense | 0.1 |
|  | p.Tyr166* | c.498C>G | Exon 5 | Nonsense | 0.6 |
|  | p.Arg169Ser | c.505C>A | Exon 5 | Missense | 0.1 |
|  | p.Arg169Cys | c.505C>T | Exon 5 | Missense | 0.2 |
|  | p.Arg169His | c.506G>A | Exon 5 | Missense | 0.1 |
|  | p.His170Arg | c.509A>G | Exon 5 | Missense | 0.1 |
|  | p.His170Gln | c. 510T>A | Exon 5 | Missense | 0.3 |
|  | **p.Arg155Valfs*40**^△^ | c.463delC | Exon 5 | Deletion | 0.1 |
|  | — | c.509+1G>A | Intron 5 | Splicing | 0.1 |
|  | — | c.510-1G>A | Intron 5 | Splicing | 0.1 |
|  | — | c.510-1G>C | Intron 5 | Splicing | 0.1 |
|  | p.Gly171Arg | c.511G>A | Exon 6 | Missense | 0.1 |
|  | p.Gln172His | c.516G>T | Exon 6 | Missense | 0.1 |
|  | p.Arg176* | c.526C>T | Exon 6 | Nonsense | 2.0 |
|  | p.Glu178Lys | c.532 G>A | Exon 6 | Missense | 0.1 |
|  | p.Glu183Gly | c.548A>G | Exon 6 | Missense | 0.1 |
|  | p.Trp187* | c.561G>A | Exon 6 | Nonsense | 0.1 |
|  | p.Trp187Arg | c.559T>C | Exon 6 | Missense | 0.1 |
|  | **p.Gly188Val**^△^ | c.563G>T | Exon 6 | Missense | 0.1 |
|  | p.His201Arg | c.602A>G | Exon 6 | Missense | 0.1 |
|  | **p.Cys203Ser**^△^ | c.607T>A | Exon 6 | Missense | 0.1 |
|  | p. Ex6-96A>G | c.611A>G | Exon 6 | Splicing | 0.1 |
|  | p.Cys217Tyr | c.650G>A | Exon 6 | Missense | 0.1 |
|  | p.Ile224Thr | c.671T>C | Exon 6 | Missense | 0.4 |
|  | **p.Leu227Val**^△^ | c.678C>G | Exon 6 | Missense | 0.1 |
|  | **p.Glu228Asp**^△^ | c.682G>T | Exon 6 | Missense | 0.1 |
|  | p.Val230Ile | c.688G>A | Exon 6 | Missense | 0.5 |
|  | p.Val230A | c.689T>C | Exon 6 | Missense | 0.1 |
|  | p.Gln232* | c.694C>T | Exon 6 | Nonsense | 0.2 |
|  | p.Phe233Leu | c.699C>A | Exon 6 | Missense | 0.2 |
|  | **p.Leu194Glufs*6**^△^ | c.580C>GA | Exon 6 | Indel | 0.1 |
|  | **p.Ser231Valfs*52**^△^ | c.690-691insG | Exon 6 | Insertion | 0.1 |
|  | — | c.707-1G>A | Intron 6 | Splicing | 0.2 |
|  | p.Arg241Cys | c.721C>T | Exon 7 | Missense | 4.6 |
|  | p.Arg241His | c.722G>A | Exon 7 | Missense | 0.4 |
|  | p.Arg241Leu | c.722G>T | Exon 7 | Missense | 0.1 |
|  | p.Leu242Phe | c.724C>T | Exon 7 | Missense | 0.2 |
|  | p.Arg243Gln | c.728G>A | Exon 7 | Missense | 17.7 |
|  | p.Arg243* | c.727C>T | Exon 7 | Nonsense | 0.1 |
|  | p.Val245Met | c.733G>A | Exon 7 | Missense | 0.1 |
|  | p.Gly247Val | c.740G>T | Exon 7 | Missense | 1.5 |
|  | p.Gly247Arg | c.739G>C | Exon 7 | Missense | 0.7 |
|  | **p.Ser250Phe**^△^ | c.749C>T | Exon 7 | Missense | 0.1 |
|  | p.Arg252Gln | c.755G>A | Exon 7 | Missense | 0.6 |
|  | p.Arg252Trp | c.754C>T | Exon 7 | Missense | 0.3 |
|  | p.Arg252Gly | c. 754C>G | Exon 7 | Missense | 0.1 |
|  | p.Arg252Pro | c.755G>C | Exon 7 | Missense | 0.1 |
|  | p.Leu255Ser | c.764T>C | Exon 7 | Missense | 0.4 |
|  | p.Gly257Val | c.770G>T | Exon 7 | Missense | 0.6 |
|  | p.Arg261Gln | c.782G>A | Exon 7 | Missense | 1.7 |
|  | p.Arg261* | c.781C>T | Exon 7 | Nonsense | 0.1 |
|  | p.Phe263Leu | c.787C>T | Exon 7 | Missense | 0.1 |
|  | p.Gln267Glu | c.799C>G | Exon 7 | Missense | 0.2 |
|  | **p.Tyr268***^△^ | c.804C>A | Exon 7 | Nonsense | 0.1 |
|  | p.Arg270Lys | c.809G>A | Exon 7 | Missense | 0.2 |
|  | p.Arg270Ile | c. 809G>T | Exon 7 | Missense | 0.1 |
|  | p.His271Arg | c.812A>G | Exon 7 | Missense | 0.1 |
|  | p.Pro275Leu | c.824C>T | Exon 7 | Missense | 0.2 |
|  | p.Met276Lys | c.827T>A | Exon 7 | Missense | 0.2 |
|  | p.Met276Arg | c.827T>G | Exon 7 | Missense | 0.1 |
|  | p.Thr278Ile | c.833C>T | Exon 7 | Missense | 0.1 |
|  | p.Glu280Lys | c.838G>A | Exon 7 | Missense | 0.4 |
|  | p.Pro281Arg | c.842C>G | Exon 7 | Missense | 0.1 |
|  | p.Arg241Profs*100 | c.722delG | Exon 7 | Deletion | 0.5 |
|  | — | c.842+1G>A | Intron 7 | Splicing | 0.2 |
|  | — | c.843-1G>A | Intron 7 | Splicing | 0.2 |
|  | — | c.842+2T>A | Intron 7 | Splicing | 1.5 |
|  | p.Val291Met | c.871G>A | Exon 8 | Missense | 0.1 |
|  | p.Ala300Ser | c.898G>T | Exon 8 | Missense | 0.1 |
|  | p.Ser303Pro | c.907T>C | Exon 8 | Missense | 0.1 |
|  | p.Ser303Profs*38 | c.907delT | Exon 8 | Deletion | 0.1 |
|  | — | c.912+1G>A | Intron 8 | Splicing | 0.1 |
|  | — | **c.912+16T>A**^△^ | Intron 8 | Splicing | 0.1 |
|  | — | c.913-7A>G | Intron 8 | Splicing | 0.2 |
|  | p.Ser310Phe | c.929C>T | Exon 9 | Missense | 0.1 |
|  | **p.Ser310Cys**^△^ | c.929C>G | Exon 9 | Missense | 0.1 |
|  | p.Gly312Val | c.935G>A | Exon 9 | Missense | 0.1 |
|  | p.Pro314Thr | c.940C>A | Exon 9 | Missense | 0.4 |
|  | p.Ala322Thr | c.964G>A | Exon 9 | Missense | 0.2 |
|  | p.Ile324Asn | c.971T>A | Exon 10 | Missense | 0.4 |
|  | p.Trp326* | c.977G>A | Exon 10 | Nonsense | 0.2 |
|  | p.Phe331Ser | c.992T>C | Exon 10 | Missense | 0.1 |
|  | **p.Ser339Phe**^△^ | c.1016C>T | Exon 10 | Missense | 0.1 |
|  | **p.Lys341Asn**^△^ | c.1023G>C | Exon 10 | Missense | 0.1 |
|  | p.Ala342Hisfs*58 | c.1024delG | Exon 10 | Deletion | 0.1 |
|  | p.Gly344Asp | c.1031G>A | Exon 10 | Missense | 0.1 |
|  | p.Gly344Ser | c.1030G>A | Exon 10 | Missense | 0.1 |
|  | p.Ala345Thr | c.1033G>A | Exon 10 | Missense | 0.1 |
|  | p.Ser349Ala | c.1045T>G | Exon 10 | Missense | 0.4 |
|  | p.Gly352Arg | c.1054G>C | Exon 10 | Missense | 0.1 |
|  | p.Gln355* | c.1063C>T | Exon 10 | Nonsense | 0.1 |
|  | — | c.1066-1G>T | Intron 10 | Splicing | 0.2 |
|  | — | c.1066-11G>A | Intron 10 | Splicing | 0.1 |
|  | — | **c.1066-13delT**^△^ | Intron 10 | Splicing | 0.1 |
|  | — | c.1066-14C>G | Intron 10 | Splicing | 0.1 |
|  | p.Tyr356* | c.1068C>A | Exon 11 | Nonsense | 4.7 |
|  | p.Cys357* | c.1071C>A | Exon 11 | Nonsense | 0.1 |
|  | **p.Pro362Ser**^△^ | c.1084C>T | Exon 11 | Missense | 0.1 |
|  | p.Pro362Thr | c.1084C>A | Exon 11 | Missense | 0.1 |
|  | p.Lys363Asn | c.1089G>T | Exon 11 | Missense | 0.2 |
|  | **p.Pro366Ala**^△^ | c.1096C>G | Exon 11 | Missense | 0.1 |
|  | p.Thr372Ser | c.1114A>T | Exon 11 | Missense | 0.1 |
|  | p.Thr372Arg | c.1115 C>G | Exon 11 | Missense | 0.1 |
|  | p.Ala373Thr | c.1117G>A | Exon 11 | Missense | 0.1 |
|  | p.Gln375Glu | c.1123C>G | Exon 11 | Missense | 0.3 |
|  | p.Thr380Met | c.1139C>T | Exon 11 | Missense | 0.2 |
|  | p.Val388Met | c.1162G>A | Exon 11 | Missense | 0.1 |
|  | p.Ser391Thr | c.1172G>C | Exon 11 | Missense | 0.1 |
|  | p.Phe392Ile | c.1174T>A | Exon 11 | Missense | 0.4 |
|  | p.Lys398= | c.1194A>G | Exon 11 | Splicing | 0.1 |
|  | p.Val399= | c.1197A>T | Exon 11 | Splicing | 6.4 |
|  | p.Arg400Thr | c.1199G>C | Exon 11 | Missense | 0.5 |
|  | p.Arg400Lys | c.1199G>A | Exon 11 | Missense | 0.2 |
|  | — | c.1199+1G>C | Intron11 | Splicing | 0.2 |
|  | — | c.1199+2T>C | Intron 11 | Splicing | 0.2 |
|  | — | c.1200-1G>C | Intron 11 | Splicing | 0.1 |
|  | — | c.1200-1G>A | Intron 11 | Splicing | 0.2 |
|  | — | **c.1200-3T>G**^△^ | Intron11 | Splicing | 0.2 |
|  | p.Ala403Val | c.1208C>T | Exon 12 | Missense | 1.0 |
|  | p.Arg408Trp | c.1222C>T | Exon 12 | Missense | 0.4 |
|  | p.Arg408Gln | c.1223G>A | Exon 12 | Missense | 0.6 |
|  | p.Arg413Pro | c.1238G>C | Exon 12 | Missense | 4.6 |
|  | p.Tyr414* | c.1242C>A | Exon 12 | Nonsense | 0.1 |
|  | p.Asp415Tyr | c.1243G>T | Exon 12 | Missense | 0.1 |
|  | p.Thr418Pro | c.1252A>C | Exon 12 | Missense | 0.6 |
|  | p.Gln419Arg | c.1256A>C | Exon 12 | Missense | 0.7 |
|  | p.Ile421Thr | c.1262T>C | Exon 12 | Missense | 0.2 |
|  | p.Gln429Lys | c.1285C>A | Exon 12 | Missense | 0.1 |
|  | p.Leu430Pro | c.1289T>C | Exon 12 | Missense | 0.2 |
|  | p.Ala434Asp | c.1301C>A | Exon 12 | Missense | 1.7 |
|  | — | c.1315+4A>G | Intron 12 | Splicing | 0.1 |
|  | — | c.1315+6T>A | Intron 12 | Splicing | 0.3 |
|  | — | c.1316-2A>C | Intron 12 | Splicing | 0.2 |
|  | **p.Leu444Phe**^△^ | c.1330C>T | Exon 13 | Missense | 0.1 |
|  | p.*453Proext*33 | c.1357delTAAAG | Exon 13 | Deletion | 0.1 |
|  | c.(168+1_169-1)_c. (352+1_353-1)del  c. (441+1_442-1)_(842+1_843-1)del | | 5’ UTR  ~E1 | Deletion | 0.6 |
|  |  |  | E3 | Deletion | 0.1 |
|  |  |  | E4~E7 | Deletion | 0.1 |
|  | c. (441+1_442-1) _c. (509+1_510-1)del | | E5 | Deletion | 0.1 |
|  | c. (441+1_442-1)_(509+1_510-1)del | | E4~E5 | Deletion | 0.2 |
| Detected | | | | | 96.6 |
| Unknown | | | | | 3.4 |
| Total | | | | | 100 |

^△^Novel alleles identified in the current study.
